# Betulinic acid enhances TGF-β signaling by altering TGF-β receptors partitioning between lipid-raft/caveolae and non-caveolae membrane microdomains in mink lung epithelial cells

**DOI:** 10.1186/s12929-016-0229-4

**Published:** 2016-02-27

**Authors:** C. L. Chen, C. Y. Chen, Y. P. Chen, Y. B. Huang, M. W. Lin, D. C. Wu, H. T. Huang, M. Y. Liu, H. W. Chang, Y. C. Kao, P. H. Yang

**Affiliations:** Department of Biological Science, National Sun Yat-sen University, Kaohsiung, 804 Taiwan ROC; Doctoral Degree Program in Marine Biotechnology, National Sun Yat-sen University and Academia Sinica, Kaohsiung, 804 Taiwan ROC; Graduate Institute of Clinical Pharmacy, College of Pharmacy, Kaohsiung Medical University, Kaohsiung, 807 Taiwan ROC; Division of Gastroenterology, Department of Internal Medicine, Kaohsiung Medical University Hospital, Kaohsiung, 807 Taiwan ROC; Center for Stem Cell Research, Kaohsiung Medical University, Kaohsiung, 807 Taiwan ROC; Division of Pathology, Department of Internal Medicine, Kaohsiung Medical University Hospital, Kaohsiung, 807 Taiwan ROC; Taiwan Ocean Research Institute, National Applied Research Laboratories, Kaohsiung, 852 Taiwan ROC

**Keywords:** Betulinic acid, Lipid-raft, Caveolae, TGF-beta

## Abstract

**Background:**

TGF-β is a key modulator in the regulation of cell proliferation and migration, and is also involved in the process of cancer development and progression. Previous studies have indicated that TGF-β responsiveness is determined by TGF-β receptor partitioning between lipid raft/caveolae-mediated and clathrin-mediated endocytosis. Lipid raft/caveolae-mediated endocytosis facilitates TGF-β degradation and thus suppressing TGF-β responsiveness. By contrast, clathrin-mediated endocytosis results in Smad2/3-dependent endosomal signaling, thereby promoting TGF-β responsiveness. Because betulinic acid shares a similar chemical structure with cholesterol and has been reported to insert into the plasma membrane, we speculate that betulinic acid changes the fluidity of the plasma membrane and modulates the signaling pathway associated with membrane microdomains. We propose that betulinic acid modulates TGF-β responsiveness by changing the partitioning of TGF-β receptor between lipid-raft/caveolae and non-caveolae microdomain on plasma membrane.

**Methods:**

We employed sucrose-density gradient ultracentrifugation and confocal microscopy to determine membrane localization of TGF-β receptors and used a luciferase assay to examine the effects of betulinic acid in TGF-β-stimulated promoter activation. In addition, we perform western blotting to test TGF-β-induced Smad2 phosphorylation and fibronectin production.

**Results and conclusions:**

Betulinic acid induces translocation of TGF-β receptors from lipid raft/caveolae to non-caveolae microdomains without changing total level of TGF-β receptors. The betulinic acid-induced TGF-β receptors translocation is rapid and correlate with the TGF-β-induced PAI-1 reporter gene activation and growth inhibition in Mv1Lu cells.

**Electronic supplementary material:**

The online version of this article (doi:10.1186/s12929-016-0229-4) contains supplementary material, which is available to authorized users.

## Background

**Fig. 1:**
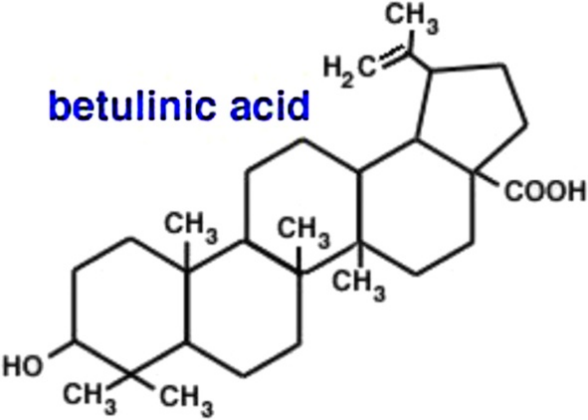
Structure of betulinic acid (BetA)

Betulinic acid (BetA) [1] is a luphane-type pentacyclic triterpenoid (Fig. 1) of plant origin that is wildly distributed in the outer bark of various tree species (eg, white-barked birch trees). BetA exhibits diverse biological activities, and antiviral, antibacterial, antioxidant, anti-inflammatory, antifibrotic, and anticancer properties [2, 3]. Antitumorigenesis is one of the most promising functions of BetA. Previous reports have indicated that BetA induces apoptosis in colorectal (DLD-1), breast (MCF7), prostate (PC-3), and lung (A549) cancer cells via the mitochondrial pathway [4–6]. In addition, past studies have reported that BetA-induced apoptosis is not associated with the activation of ligand/receptor systems such as CD95, and does not involve p53 [1]. In BetA-induced apoptosis, the perturbation of mitochondrial function (eg, loss of mitochondrial permeability transition and the production of reactive oxygen species) precedes other key features of apoptosis such as the activation of the caspase cascade and nuclear fragmentation [7, 8]. Recent studies have indicated that BetA also modulate signaling through members of the TGF-β superfamily, which regulates inflammatory and immune responses, cell growth, differentiation, and apoptosis [9, 10]. However, the underlying mechanisms regarding the effects of BetA in TGF-β signaling are poorly understood.

TGF-β is a family of 25-kDa disulfide-linked dimeric proteins. It has 3 members in mammals (TGF-β_1_, TGF-β_2_, and TGF-β_3_,), which share approximately 70 % of the sequence homology [11, 12]. TGF-β exhibits bifunctional growth regulation; it inhibits the growth of most cell types, including epithelial cells, endothelial cells, and lymphocytes, and stimulates the proliferation of mesenchymal cells such as fibroblasts [11, 12]. In epithelial cells, TGF-β inhibits cell proliferation, induces apoptosis, and mediates differentiation, implying that this signaling pathway engages in tumor-suppressing activities in epithelial tumors [13, 14]. However, TGF-β promotes invasive and metastatic activities in late-stage tumor progression, implying that TGF-β can paradoxically play opposing roles in human cancers, and this is seemingly dependent on the cancer stage. In addition to its growth regulatory activities, TGF-β exhibits other biological activities, including the regulation of extracellular matrix synthesis, chemotaxis, angiogenesis, and the differentiation of several cell lineages. It has been implicated in numerous pathophysiological processes including wound repair, tissue fibrosis, immunosuppression, and morphogenesis [15]. The primary biological activities of TGF-β are mediated by specific cell surface receptors type I and type II (TβR-I and TβR-II, respectively). TGF-β exerts its effects on cells by binding to TβR-II, which induces the recruitment of TβR-I with the subsequent activation of the receptor complex. Smad2 and Smad3 are the direct substrates of the activated TGF-β receptor complex. After stimulation, the Smad complex translocates into the nucleus, where it functions as a member of different transcription factor complexes that regulate the expression of various genes [12, 13, 16].

We have previously reported that suppressed TGF-β responsiveness in the aortic endothelium plays a critical role in the pathogenesis of atherosclerosis in hypercholesterolemic animals [17, 18]. A high concentration of cholesterol in the culture medium suppresses TGF-β responsiveness in cultured cells, including in endothelial cells, by inducing the accumulation of cell-surface TGF-β-TGF-β receptor complexes in the lipid rafts/caveolae of the plasma membrane, facilitating the rapid degradation of these complexes, thereby attenuating TGF-β-stimulated signaling and related responses [17–19]. This effect of cholesterol is believed to be mediated by the increasing formation or stabilization of the lipid rafts/caveolae, presumably through the direct insertion of cholesterol into the plasma membranes of target cells [17, 18]. Lipid raft/caveolae are thought to form platforms collecting assemblies of proteins involved in many key cellular functions, including signal transduction, membrane fusion, cytoskeleton organization, lipid sorting, protein trafficking, and the localization and activities of specific membrane channels [20–22]. Because BetA shares a similar chemical structure with cholesterol, and was reported to insert into the plasma membrane [23], it is rational to speculate that BetA changes the fluidity of the plasma membrane and modulates the signaling pathway associated with membrane microdomains. We speculate that BetA modulates TGF-β responsiveness by changing the partitioning of the TGF-β receptor between the microdomains of the lipid raft/caveolae and non-caveolae on the plasma membrane.

Because the TGF-β signaling pathway is a key mediator that controls proliferation and inflammation, and BetA has antitumor and anti-inflammation properties, this study investigates the effect of BetA on TGF-β signaling, and we attempt to elucidate the mechanisms involved. We found that BetA activates TGF-β receptors by moving them from lipid raft to non-raft membrane microdomains, as perceived by the enhancement in TGF-β-specific reporter gene activity, sucrose gradient fractionation of the plasma membrane, fibronectin protein levels, Smad2/3 phosphorylation, nuclear translocation, and TGF-β-induced growth inhibition.

## Methods

### Materials

Dulbecco’s modified Eagle's medium (DMEM), phenylmethanesulfonyl fluoride (PMSF), Betulinic acid [(3b)-3-hydroxy-lup-20(29)-en-28-oic acid], bovine serum albumin (BSA), and peroxidase-conjugated anti-rabbit IgG were obtained from Sigma (St. Louis, MO). The pre-stained protein ladder (64, 49, 37, 26, and 20 kDa) and fetal calf serum (FCS) was obtained from Invitrogen (Carlsbad, CA). TGF-β_1_ was purchased from Austral Biologicals (San Ramon, CA). The polyclonal antibodies against early endosome antigen 1 (EEA1), transferrin receptor (TfR), Smad2/3, Caveolin-1, flotillin-2, epidermal growth factor receptor (EGFR), TβR-I, TβR-II, and HA-probe were obtained from Santa Cruz (Santa Cruz, CA). The rabbit polyclonal antibody to phospho-Smad2 was purchased from Cell Signaling (Boston, MA).

### Cell culture

Mink lung epithelial (Mv1Lu) cells and Mv1Lu cell stably express plasminogen activator inhibitor-1 (PAI-1) luciferase promoter were kindly provided by Dr. Jung San Huang (Saint Louis University, Saint Louis, MO). The Mv1Lu cells were cultured in DMEM supplemented with 10 % FCS, 1 % penicillin, and streptomycin (pH 7.4). The cells were seeded in tissue culture plates (Falcon, Bedford, MA, USA) and incubated at 37 °C in a humidified atmosphere of 5 % CO_2_. The Mv1Lu cells were subcultured twice per week through trypsinization in a 0.25 % trypsin-EDTA solution after washing with Ca^2+^-Mg^2+^-free saline.

### Treatment of Mv1Lu cells with TGF-β after preincubation with BetA

The Mv1Lu cells were grown in 12-well plastic plates (4 × 10^5^ cells/mL with 1 mL/well 10 % FCS-DMEM) for 24 h in a humidified CO_2_ incubator at 37 °C. Afterward, the medium was replaced with fresh 1 % FCS-DMEM. The cells were subsequently preincubated with BetA for 1 h at 37 °C. Next, TGF-β was added to the medium, and incubation continued for an additional 48 h; the cultures were then washed twice with cold PBS, and harvested for Western blot analysis. To observe Smad2/3 phosphorylation, the cells in the 12-well plastic plates were preincubated with BetA for 1 h at 37 °C. Afterward, TGF-β1 was added to the medium, and incubation continued for 30 min.

### Western blot analysis

The cell lysates of Mv1Lu cells (approximately 50 μg protein) were subjected to 10 % SDS-PAGE under reducing conditions, and then electrotransferred to PVDF membranes. After being incubated with 5 % nonfat milk in Tris-buffered saline plus Tween 20 (TBST) (50 mM Tris–HCl, pH 8.0, 150 mM NaCl, and 0.05 % Tween 20) for 1 h at room temperature, the membranes were incubated further with specific polyclonal antibodies to TβR-I and TβR-II in TBST/non-fat milk at 4 °C for 18 h, and washed 3 times with TBST for 10 min each. The bound antibodies were detected using peroxidase-conjugated anti-rabbit IgG and visualized using the ECL system (ImageQuant).

### Luciferase activity assay

The Mv1Lu cells were transiently transfected with fibronectin [24] and collagen [25] luciferase promoter plasmids through electroporation. The cell suspension was mixed with 14 μg/mL of plasmid DNA (and renilla control plasmid) before it was transferred into an electroporation cuvette (0.4 cm gap, Bio-Rad, Hercules, CA) and pulsed (950 μF, 250 V, Gene Pulser II, Bio-Rad). After electroporation, the cells were seeded in 24-well cluster plates (Corning), and growth continued for an additional 24 h. The Mv1Lu cells stably express luciferase reporter gene driven by the PAI-1 promoter (MLECs – Clone 32), grown to near confluence on 24-well plates, were treated with different concentrations of BetA, with or without varying concentrations of cholesterol at 37 °C for 1 h. The treated cells were further incubated with 50 pM TGF-β1 at 37 °C for 6 h, and lysed in 100 μL of a lysis buffer (Promega). The cell lysates (approximately 20 μg of protein) were then mixed with a D-luciferin (Gold Biotechnology, St. Louis, MO) assay buffer and assayed using the luminometer (Titertek-Berthold, Pforzheim, Germany). The luciferase count was corrected for renilla activity, and a relative increase in corrected luciferase count was calculated versus the controls. A constitutively active ALK-5 (caALK-5) construct was cotransfected at a concentration of 1.2 μg/mL in the indicated experiments.

### Immunofluorescent detection of Smad2/3

Cells were grown on 24-mm round coverglass (Paul Marienfeld). After 1 h of serum starvation and 1 h of pretreatment with 5 μg/mL of BetA or the vehicle, cells were stimulated with TGF-β1 (20 pM) for 30 min. They were then washed with phosphate buffered saline (PBS) and fixed in cold methanol for 15 min. Afterward, the cells were blocked with 5 % goat serum (Dako) in 1 % BSA/PBS. After incubation with mouse-anti-Smad2/3 (H-2; Santa Cruz Biotechnology) at 1:100 dilution in 1 % BSA/PBS for 18 h at 4 °C, the cells were incubated with donkey anti-mouse-FITC (Gene Tex) at room temperature for 1 h. The coverglass was mounted with a slow-fade gold antifade reagent and DAPI (Invitrogen). Photomicrographs were taken using a Zeiss Axio Observer Z1 microscope equipped with a Photometrics HQ2 camera.

### Immunofluorescent confocal microscopy

The Mv1Lu cells were placed in a 24-mm coverglass and transiently transfected with TβR-II-HA plasmid (0.4 μg) by using lipofectamin 2000 (Invitrogen) in accordance to the manufacturer protocol. The transfected cells were pretreated with 5 μg/mL of BetA at 37 °C for 1 h, and then incubated with 100 pM of TGF-β1 for 30 min. After TGF-β1 stimulation, the cells were fixed in methanol at −20 °C for 15 min, washed with PBS, and then blocked using 0.2 % gelatin in PBS for 1 h. Cells were incubated overnight at 4 °C in a humidified chamber with a goat antibody against HA-probe (F-7, Santa Cruz Biotechnology) and a rabbit antibody against caveolin-1 (N-20, Santa Cruz Biotechnology) at 1:100 dilutions. After extensive washing, the cells were incubated with Rhodamine-conjugated donkey anti-goat antibody and FITC-conjugated mouse anti-rabbit antibody at a 1:50 dilution for 1 h. Images were acquired using a Leica TCS SP confocal microscope (Leica Microsystems Ltd., Heidelberg, Germany). The measurements of the colocalization rate were analyzed using a Leica Application Suite.

### Separation of lipid-raft and non-lipid raft microdomains of plasma membranes by sucrose density gradient ultracentrifugation

Sucrose density gradient analysis was performed at 4 °C as described previously [26]. In brief, cells were grown to near-confluence in 100-mm dishes (5–10 × 10^6^ cells per dish). Cells were incubated with BetA (5 μg/mL) or cholesterol (25 μg/mL) at 37 °C for 4 h. After 2 washes with ice-cold PBS, the cells were scraped in 0.85 mL of 500 mM sodium carbonate (pH 11). Homogenization was performed with 10 strokes of a tightfitting Dounce homogenizer, followed by three 15-s bursts of an ultrasonic disintegrator (Qsonica, Newtown, CT, USA) to disrupt the cell membranes. The homogenates were adjusted to 45 % sucrose by adding 0.85 mL of 90 % sucrose in 25 mM 2-(*N*-morpholino) ethanesulfonic acid (pH 6.5) and 0.15 M NaCl (MBS), and placed at the bottom of an ultracentrifuge tube. A discontinuous sucrose gradient was generated by overlaying 1.7 mL of 35 % sucrose and of 5 % sucrose in MBS on top of the 45 % sucrose solution, and it was subsequently centrifuged at 40 000 rpm for 16–20 h in a P50S2 rotor (Himac, Tokyo, Japan). Ten 0.5-mL fractions were collected from the top of the tube, and a portion of each fraction was analyzed through SDS-PAGE, followed by Western blot analysis using antibodies to TβRI (ALK-5), TβRII, TfR-1, EEA-1, flotillin-2, EGFR, and caveolin-1. The relative amounts of TβRI, TβRII, TfR-1, and caveolin-1 on the blot were quantified through densitometry. The protein recovery and localization of caveolin-1, flotillin-2, and TfR-1 (fractions 4 to 5 and 8 to 10, respectively) did not change significantly with any of the treatment protocols (Fig. 8). An HA-tagged TβRII (TβRII-HA) construct was transfected at a concentration of 0.5 μg/mL in the indicated experiments.

### MTT viability assay

The effect of BetA on TGF-β-induced growth inhibition was determined using an MTT (3-[4,5-dimethylthiazol-2-yl]-2,5-diphenyl tetrazoliumbromide) assay. In brief, the cells were plated at 1× 10^4^ cells per well in 150 μL of a culture medium containing 1 % FCS and the desired concentrations of BetA, before they were diluted with the culture media to achieve final concentrations ranging from 0.5 to 5 μg/mL. The EtOH concentration remained within the maximum permissible concentration of 0.01 % in both the control and treated samples. After pretreatment with BetA, the cells were treated with TGF-β, and incubation continued for 30 h at 37 °C in a humidified incubator, after which cell viability was determined. Afterward, 50 μL of MTT (4 μg/mL in PBS stock, diluted to a working strength of 1 μg/mL with the media) was added to each well and incubated for 2 h. After the medium was carefully removed, 0.1 mL of DMSO was added to each well, and the plates were shaken. Absorbance was recorded on a microplate reader, at a wavelength of 570 nm. The effect of BetA on growth inhibition was assessed as the percentage of cell viability, where the vehicle-treated cells were considered 100 % viable.

### Statistical analysis

The data represent the means ± standard deviation (SD). Group means were compared by one-way ANOVA (analysis of variance), followed by Tukey’s procedure for multiple comparisons if necessary, using statistical software program SPSS ver. 17.0 for Windows (SPSS, Chicago, IL, USA). In all comparisons, a value of p < 0.01 was considered to indicate a statistically significant difference.

## Results

### BetA enhances TGF-β-induced transcriptional responsive

**Fig. 2:**
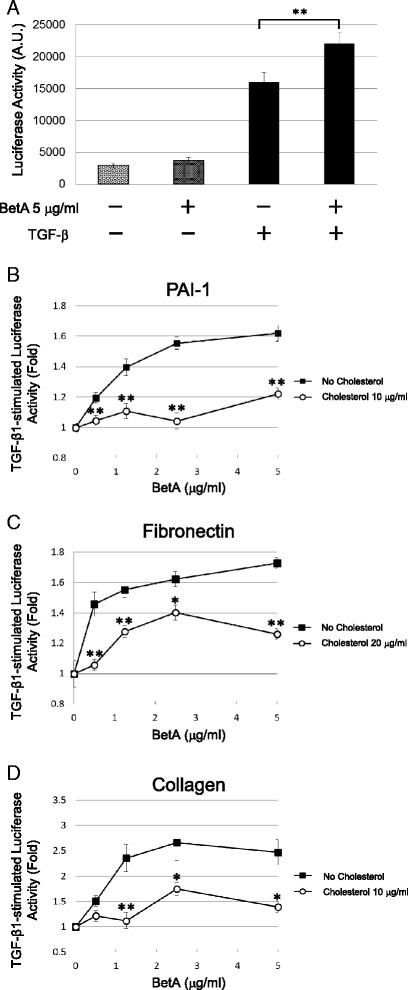
BetA enhances the transcriptional response stimulated by TGF-β in Mv1Lu cells. Cells stably expressing the PAI-1 luciferase reporter plasmid exhibited a 6-fold increase of the luciferase activity after stimulation with 100 pM TGF-β and the TGF-β-stimulated luciferase activity was enhanced by BetA in a concentration-dependent manner (**a**) and (**b**). However, the BetA-enhanced TGF-β-stimulated PAI-1 luciferase activity was diminished in the presence of cholesterol (**b**). Cells transiently transfected with the fibronectin (**c**) and collagen (**d**) luciferase reporter plasmids were treated with 100 pM TGF-β and increasing concentrations of BetA and/or cholesterol. BetA also enhanced the TGF-β-stimulated luciferase activity driven by the promoters of fibronectin (**c**) and collagen (**d**). The presence of cholesterol inhibited these BetA-enhanced luciferase activities (b, **c**, and **d**). The data bar represents the mean ± S.D. ** and * indicate the significant different between cells treated with or without BetA (**a**), or lower than that in cells treated without cholesterol in the same concentration of BetA group (**b**, **c**, and **d**) (*: *P* < 0.05, **: *P* < 0.01)

**Fig. 3:**
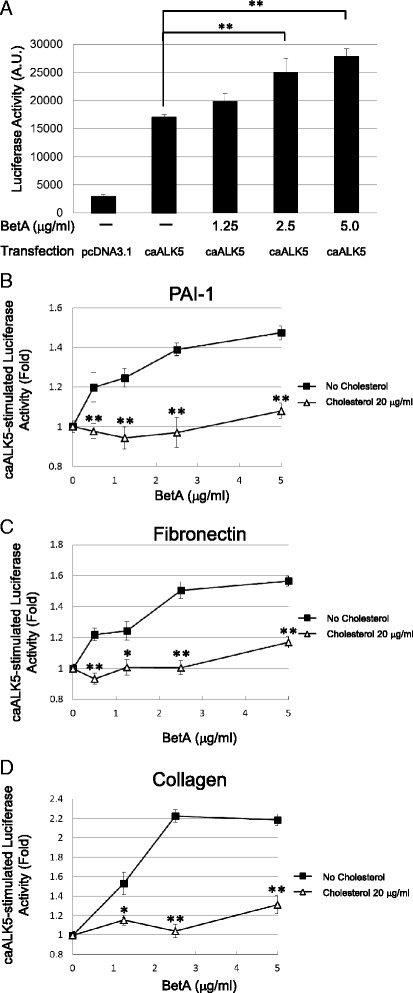
BetA enhances the TGF-β response downstream of ALK-5 in Mv1Lu cells. Cells stably expressing the PAI-1 luciferase promoter were transiently transfected with caALK-5 or pcDNA3.1 (as a control). These transfected cells exhibited a potent luciferase activity in the absence of exogenously added TGF-β. BetA appeared to enhance caALK-5-stimulated PAI-1 (**a** and **b**), fibronectin (**c**), and collagen (**d**) promoter luciferase expression in a concentration-dependent manner. Cholesterol treatment suppressed the BetA-enhanced luciferase activity. The data bar represents the mean ± SD from four different analyses. * and **Significantly higher than that in cells treated without BetA (**a**) or lower than that in cells treated without cholesterol (**b**, **c**, and **d**) (*: *P* < 0.05, **: *P* < 0.01)

The Mv1Lu cells were selected to examine the effect of BetA on TGF-β signaling. The Mv1Lu cell is a model cell line for TGF-β investigation, and exhibits a strong dosage-dependent responsiveness to TGF-β, as determined by Mv1Lu cells stably expressing the luciferase reporter gene driven by the PAI-1 promoter. TGF-β (100 pM) induced luciferase activity by approximately 6-fold in Mv1Lu cells (Fig. 2a). The TGF-β-dependent luciferase activity was enhanced by BetA treatment in a concentration-dependent manner, start with an approximate 20 % increase in 0.5 μg/mL BetA-treated cells, and reaching roughly 60 % at 5 μg/mL of BetA (Fig. 2b). The BetA also enhanced the TGF-β-induced transcriptional activation of fibronectin and collagen, as determined by transient transfection with the fibronectin and collagen luciferase promoter constructs (Fig. 2c and d, respectively). This study proposed that BetA enhances TGF-β responsiveness by moving TGF-β receptors out of the lipid raft microdomain in the plasma membrane. To find supporting evidence of this hypothesis, the cells were treated with 10 μg/mL or 20 μg/mL of cholesterol, which has been reported as a critical component of lipid raft microdomains. Our previous reports have indicated that adding cholesterol to cultured cells could induce recruitment of TGF-β receptors into the lipid raft membrane microdomain and facilitate receptor degradation [17, 18]. As shown in Fig. 2, cholesterol prevented the BetA-enhanced transcriptional activation of PAI-1, fibronectin, and collagen induced by TGF-β (Fig. 2b, c, and d, respectively). Cholesterol and BetA did not affect the basic reporter gene activity, indicating that the induced TGF-β response was affected, not the luciferase activity or cellular activity itself. We did not observe any cell death from treatment with either BetA or cholesterol, as determined by MTT assay and trypan blue cell exclusion assays (data not shown). To further assess whether BetA acts on ligand binding and subsequent active receptor complex formation or on downstream TGF-β signaling, Mv1Lu cells were transiently transfected with caALK-5, which resulted in an 8-fold induction of PAI-1 promoter activity without TGF-β stimulation (Fig. 3a). The BetA enhanced caALK-5-stimulated transcriptional activities, including PAI-1, fibronectin, and collagen (Fig. 3b, c, and d, respectively), in a concentration-dependent manner. Cholesterol treatment suppressed the BetA-specific luciferase activities exclusively, not the basal luciferase activities induced by caALK-5*.* This result implies that BetA and cholesterol affect the components of the TGF-β receptor-Smad signaling pathway, rather than altering ligand binding to TGF-β receptors.

### BetA enhances TGF-β-induced Smad2 phosphorylation and nuclear translocation

**Fig. 4:**
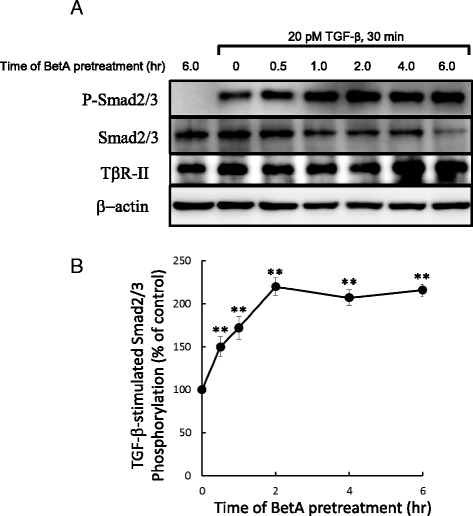
BetA enhances TGF-β-induced Smad2 phosphorylation and nuclear translocation in Mv1Lu cells. Cells were pretreated with BetA, for 0, 0.5, 1, 2, 4, and 6 h, and then further incubated with 100 pM TGF-β for 30 min. The P-Smad2 and total Smad2 levels in the cell lysates were analyzed by immunoblotting. The relative level of P-Smad2 (P-Smad2/Smad2) was estimated. A representation of 2 analyses is shown (**a**). The quantitative analysis of the immunoblots is shown (**b**). The relative level of P-Smad2 in cells treated only with TGF-β was taken as 100 % of TGF-β-stimulated Smad2 phosphorylation. The data from three different analyses represents the mean ± SD. **Significantly higher than that in cells treated with TGF-β alone (*P* < 0.01)

**Fig. 5:**
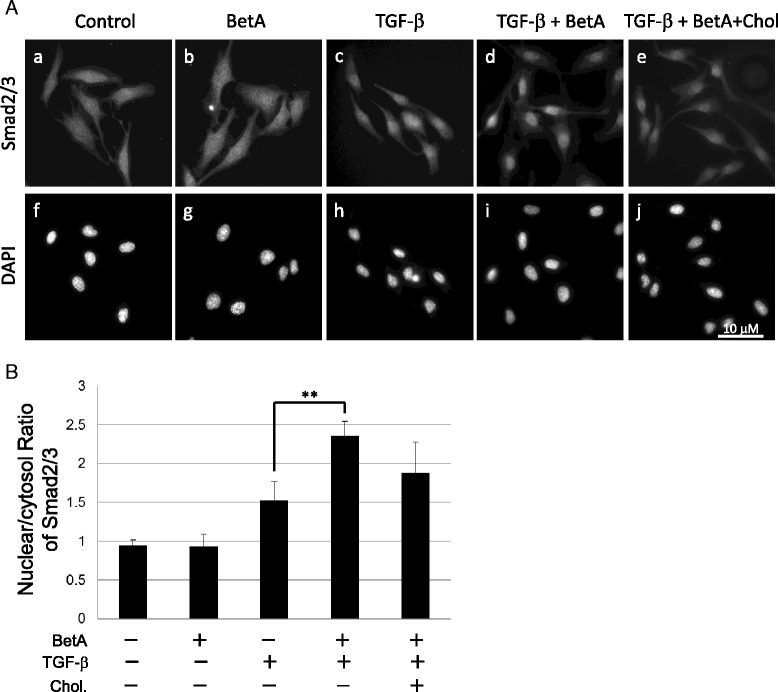
BetA increases the TGF-β-induced nuclear translocation of Smad2 in Mv1Lu cells. After 1 h of incubation of cells with 5 μg/mL BetA followed by 30 min of treatment with 20 pM TGF-β, the cells were fixed, subjected to immunofluorescence staining with primary antibodies against Smad2 as well as secondary antibodies linked to FITC, and mounted with a DAPI-containing medium. FITC fluorescence represents P-Smad2 staining (A, panels a-e), whereas the nuclei were subjected to DAPI staining (A, panels f-j). The nuclear/cytoplasm ratio of the Smad2 level was determined among 100 cells in 10 different fields, and were quantified using Image J (NIH, Bethesda, MD) (B). Values are expressed as the mean ± SD (*n* = 3). **The group of BetA/ TGF-β co-treatment significantly higher than that in cells treated with TGF-β only (*P* < 0.01)

Because cholesterol is a critical structural component of lipid rafts and caveolae [27, 28] and shares a similar chemical structure with BetA, treatment of cells with BetA may modulate TGF-β-stimulated signaling and cellular responses by altering the structure and function of lipid rafts/caveolae. To test the effect of BetA on TGF-β-induced signaling, we determined the effect of BetA treatment on TGF-β-stimulated Smad2 phosphorylation and nuclear translocation, both of which are key signaling events leading to TGF-β responsiveness [16, 29, 30]. As shown in Fig. 4a and b, BetA effectively enhanced Smad2 phosphorylation stimulated by TGF-β in a time-dependent manner in Mv1Lu cells. After 1 h of BetA pretreatment, Smad2 phosphorylation increased by 75 %. At 2 h of pretreatment, BetA enhanced Smad2 phosphorylation by over 100 %. To determine the effect of BetA on Smad2 nuclear translocation, we performed immunofluorescent staining using the anti-Smad2/3 antibody and nuclear 4′,6-diamidine-2-phenylindole (DAPI) staining. As shown in Fig. 5A, BetA enhanced TGF-β-induced Smad2 nuclear translocation (Fig. 5Ad versus Fig. 5Ac). After counting the cells that underwent Smad2 nuclear localization from 3 separate experiments, we found that TGF-β-induced Smad2 nuclear translocation in all of the treated cells, whereas BetA enhanced Smad2 nuclear translocation in 70 ± 5 % of these cells (Fig. 5B). In the experiments with BetA alone and the vehicle (0.01 % EtOH), the cells did not exhibit any nuclear translocation (Figs. 5Aa and Ab, respectively). Overall, these results imply that BetA treatment enhances TGF-β1-induced signaling.

### TGF-β1-induced fibronectin expression is promoted by BetA

**Fig. 6:**
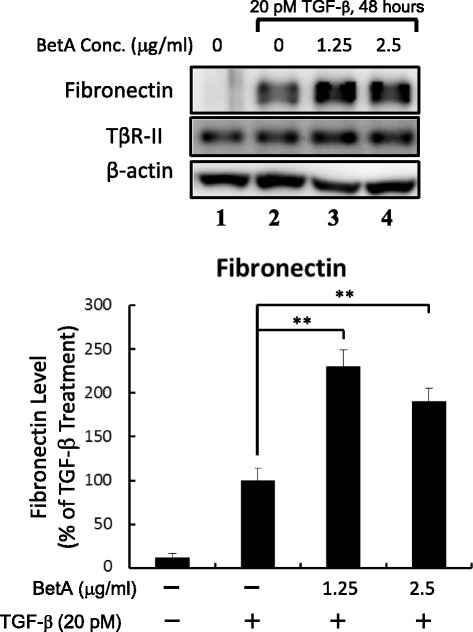
BetA promotes TGF-β-induced fibronectin expression. Cells were incubated with 1.25 μg/mL and 2.5 μg/mL of BetA, and then treated with 100 pM TGF-β for 48 h. Whole-cell extracts were prepared and analyzed by Western blot analysis using an antibody against fibronectin (*top*). The quantitative data from three analyses is shown as mean ± SD (*bottom*). **The group of BetA/TGF-β co-treatment significantly higher than that in cells treated with TGF-β only (*P* < 0.01)

One biological activity of TGF-β is the transcriptional activation of gene coding for extracellular matrix (ECM) proteins, which is a crucial event in wound healing, tissue repair, and cancer progression in adult tissue [31, 32]. During prolonged treatment, TGF-β successively induces epithelial-mesenchymal transition differentiation with an increased expression of ECM proteins, including fibronectin in epithelial cells [33, 34]. This transcriptional activation is mediated by the Smad2/3 signaling pathway. To define the effect of BetA on TGF-β responsiveness, we determined that of BetA on TGF-β-induced fibronectin expression in cells by using an ECL system for western blotting. As shown in Fig. 6, the treatment of Mv1Lu cells with BetA increased TGF-β-induced fibronectin expression: at 1.25 μg/mL, BetA enhanced fibronectin expression by approximately 95 % compared with TGF-β alone (lane 3 versus lane 2) in Mv1Lu cells. At 2.5 μg/mL of BetA treatment, we found a slight decrease in fibronectin expression compared with 1.25 μg/mL of BetA treatment, which may be due to other effects from long-term BetA treatment.

### TGF-β-induced Mv1Lu cell growth inhibition is enhanced by BetA

**Fig. 7:**
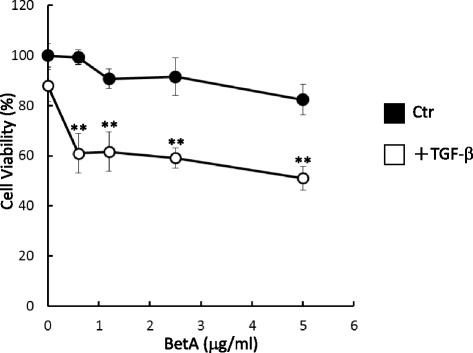
BetA enhances TGF-β-induced cell death in Mv1Lu cells. Cells were pretreated with BetA for 4 h and stimulated with 20 pM of TGF-β for 30 h. The MTT (cell viability) assay showed that BetA enhanced TGF-β-induced cell death in a concentration-dependent manner after 30 h of exposure to BetA. Data represents the mean ± SD (*n* = 3). **Significantly lower than that in cells treated with TGF-β alone (*P* < 0.01)

Another prominent biological activity of TGF-β is the growth inhibition of numerous cell types [12, 16, 30, 35–37]. If BetA enhances TGF-β responsiveness, it should augment TGF-β growth inhibition, which is also mediated by the Smad2/3 signaling pathway. To verify this hypothesis, we pretreated Mv1Lu cells with increasing concentrations (as indicated) of BetA at 37 °C for 4 h, and then further incubated them with 20 pM TGF-β at 37 °C for 30 h. In order to show the TGF-β-induced growth inhibition enhanced by BetA, we used a low concentration (20 pM) of TGF-β in these experiments. In the absence of BetA, 20 pM of TGF-β inhibited Mv1Lu cell growth by approximately 10 % (the first closed circle on the left side in Fig. 7). In the presence of BetA (0.6 to 5 μg/mL), TGF-β_1_-induced growth inhibition increased by 40 % (the closed circles in Fig. 7). In this study, none of the BetA concentrations used affected cell growth and viability significantly (the open circles in Fig. 7). The control was treated with 0.01 % EtOH without TGF-β (the first open circle from the left side in Fig. 7).

### BetA increases TGF-β receptor accumulation in the non-caveolae microdomain

**Fig. 8:**
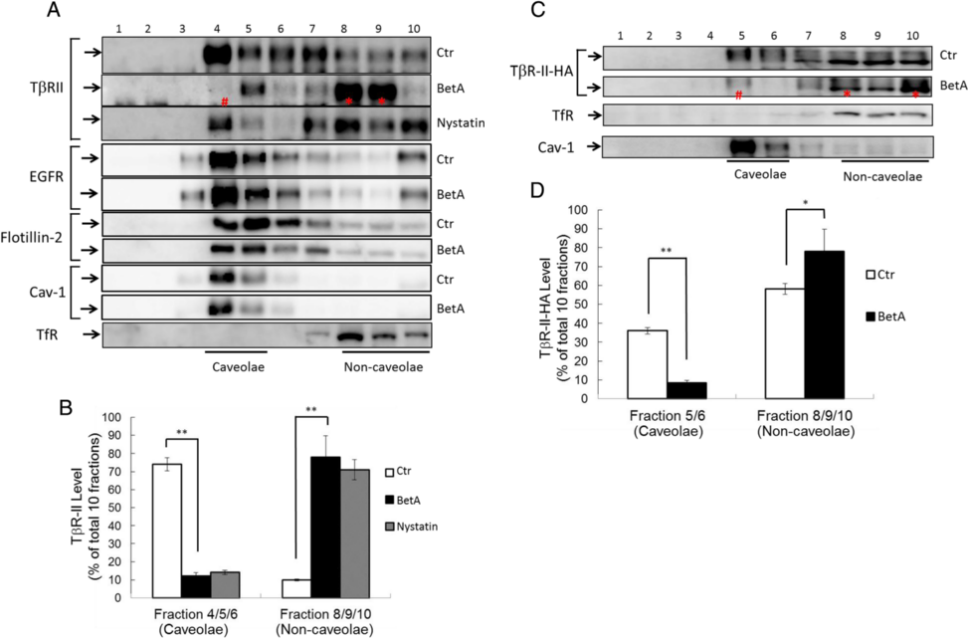
Sucrose density gradient analysis of TβR-II (**a**, **b**) and TβR-II-HA (**c**, **d**) in the plasma membrane of Mv1Lu cells and Mv1Lu cells transiently transfected with TβR-II-HA plasmid after treatment with BetA. Mv1Lu cells (**a**, **b**) or Mv1Lu cells transiently expressing TβR-II-HA (**c**, **d**) were pretreated with or without 5 μg/mL of BetA at 37 °C for 4 h. The cell lysates from these treated cells were subjected to sucrose density gradient ultracentrifugation. The sucrose gradient fractions were then analyzed by Western blot analysis using anti-TβR-II, anti-HA probe, anti-EGFR, anti-flotillin-2, anti-TfR-1, and anti-caveolin-1 antibodies. The arrow indicates the locations of TβR-II, TfR-1, and caveolin-1. Fractions 4 and 5 contained lipid rafts/caveolae, whereas fractions 8, 9, and 10 were non-lipid raft fractions. Treatment with BetA alone did not affect the total amounts of TβR-II, TβR-II-HA, and other cell proteins (Additional file 1, Fig. 1). The * symbol indicates the increased amount of TβR-II in the fraction compared with that of the untreated control. The # indicates the decreased amount of TβR-II in the fraction compared with that of the untreated control. BetA treatment induced TβR-II (or TβR-II-HA) translocation from lipid rafts/caveolae to non-lipid raft microdomains. However, BetA treatment did not alter EGFR distribution between lipid rafts/caveolae and non-lipid raft microdomains. The level of TβR-II (**a**) or TβR-II-HA (**c**) in lipid rafts/caveolae and non-lipid raft microdomains was determined. The quantitative data from three analyses is shown (**b**, **d**) as the mean ± SD (*n* = 3). *, ** Significantly lower or higher than that in cells treated without BetA (control) (*: *P* < 0.05, **: *P* < 0.01) (**b**, **d**)

**Fig. 9:**
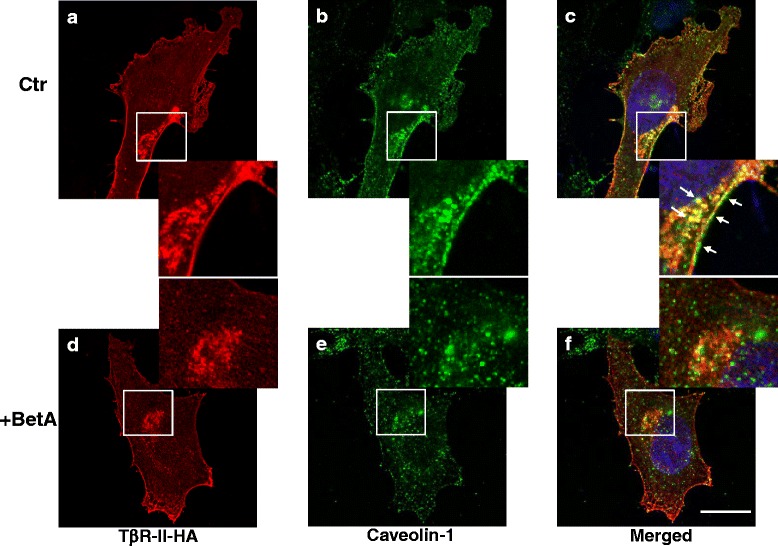
The immunofluorescence localization of TβR-II-HA and caveolin-1 in Mv1Lu cells treated with and without BetA. The Mv1Lu cells transiently expressing TβR-II-HA were treated with or without 5 μg/mL of BetA at 37 °C for 1 h. The cells were then fixed with cold methanol and incubated with a mouse antibody against HA (panels **a** and **b**) and a rabbit antibody against caveolin-1 (panels **b** and **e**), followed by incubation with Rhodamine-conjugated donkey anti-mouse antibody or FITC-conjugated goat anti-rabbit antibody. Fluorescence in cells was examined using a confocal microscope: Bar, 20 μm. The white arrows indicate the colocalization of TβR-II-HA and caveolin-1 at the cell surface and endocytic vesicle (panel **c**, enlarged picture in lower right corner.) before BetA treatment. (panel **f**, enlarged picture in upper right corner.) BetA treatment reduces the colocalization of TβR-II-HA and caveolin-1

We previously reported that TGF-β responsiveness is determined by the localization of TβR-I and TβR-II in the lipid raft/caveolae and non-caveolae microdomains of the plasma membranes [17–19, 26]. To test the effect of BetA on the plasma microdomain localization of TGF-β receptors, we analyzed the lipid raft and non-lipid raft localization of TβR-II in the plasma membrane of untreated cells or cells treated with BetA (5 μg/mL) by conducting sucrose density gradient ultracentrifugation analysis followed by western blotting. As shown in Fig. 8a, TβR-II was present in both the non-caveolae (fractions 8 to 10) and lipid raft/caveolae fractions (4 and 5), which contained transferrin receptor 1 (TfR-1) and caveolin-1, respectively. After BetA treatment, TβR-II was found to be enriched in the non-caveolae fractions (8 and 9) of the plasma membrane in Mv1Lu cells compared with the same fractions before BetA treatment (Fig. 8, BetA versus control). Treatment with nystatin, a cholesterol-depleting agent, also induced TβR-II translocation from the lipid raft/caveolae to non-caveolae microdomain. We confirmed that BetA plays a similar role to nystatin in TGF-β receptor translocation. Both BetA and nystatin moved the TGF-β receptor from fractions 4 and 5 (lipid raft) to fractions 8 to 10 (non-lipid raft) (Fig. 8a BetA versus nystatin). As shown in Fig. 8c, Mv1Lu cells transiently expressing HA-tagged TβR-II were incubated in control media or media containing 5 μg/mL of BetA, and were processed for sucrose gradient fractionation and western blotting with the anti-HA probe antibody. The BetA treatment induced TβR-II-HA translocation from lipid raft to non-lipid raft microdomains. To test whether BetA changes the membrane localization of other types of growth factor receptors, we examined EGF receptor localization in BetA-treated cells. The BetA treatment did not alter the membrane localization of EGF receptors (Fig. 8a). To further test BetA-induced TGF-β receptor translocation in the plasma membrane, Mv1Lu cells transiently expressing TβR-II-HA were treated with or without BetA, and were analyzed trough immunofluorescence microscopy by using antibodies against HA-probe and caveolin-1. As shown in Fig. 9, in the control experiment, TβR-II-HA (depicted in red) and caveolin-1 (green) were colocalized and accumulated in lipid rafts (Fig. 9c). After 30 min of BetA treatment, BetA reduced the colocalization of TβR-II-HA and caveolin-1 (Fig. 9f). Combined with the findings shown in Figs. 8 and 9, these results imply that cell treatment with BetA and nystatin increased the accumulation of TβR-II in non-caveolae microdomains, and presumably increased endosomal signaling [18, 26, 38], resulting in enhanced TGF-β responsiveness.

## Discussion and conclusion

**Fig. 10:**
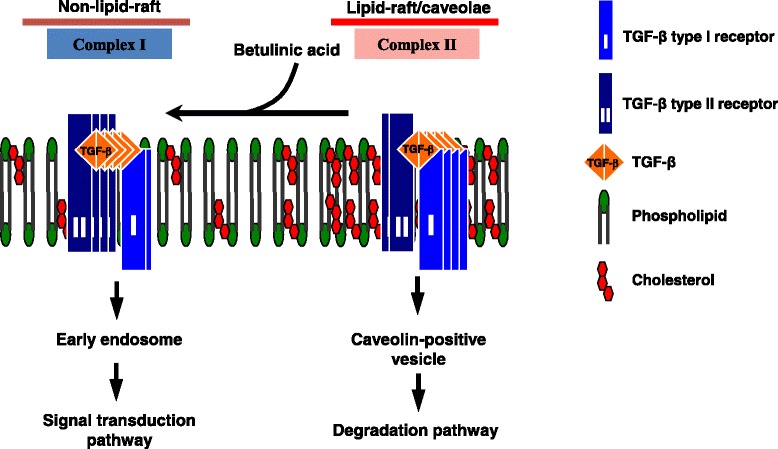
A model for the BetA effect on TGF-β partitioning between lipid rafts/caveolae- and clathrin-mediated endocytosis. Two major TβR-I-TβR-II complexes (Complexes I and II) are present on the cell surface. Complexes I and II are localized mainly in the non-lipid raft and lipid raft/caveolae microdomains, respectively, of the plasma membrane. BetA treatment results in decreased formation, or the disruption of the lipid raft, and subsequently moves TβR-I-TβR-II to non-lipid raft microdomains where TGF-β binding to the receptors induces Smad2 signaling, leading to cellular responses

Triterpenoids are studied extensively for their potential use as anticancer and antifibrotic agents [39–45], and BetA is one of the most promising compounds in this class. Several proteins and pathways are clearly targeted by BetA, but none of these individual targets provide a satisfactory and comprehensive view of the overall mechanism. The BetA compound has been shown to inhibit aminopeptidase N, an enzyme involved in the regulation of angiogenesis that is overexpressed in several cancers [46, 47]. Moreover, one study demonstrated that BetA blocks the catalytic activity of topoisomerase I by abrogating the interaction of the enzyme and the DNA substrate [48]. Another important target is the NFκB pathway, a pro-inflammatory and pro-survival node in which a transcription factor regulates many cell cycle, differentiation, and apoptosis genes; this pathway is constitutively active in many human cancers [49, 50]. The BetA suppresses NFκB activation by inhibiting IκBα kinase and p65 phosphorylation [51]. Although PPARγ, aminopeptidase N, IκBα Kinase, and NFκB are the direct targets of BetA, they still cannot account for all of its known biological activities. Because BetA is similar to cholesterol in structure, it is tempting to speculate that BetA may be intercalated into lipid rafts and might modulate their membrane fluidity like other cholesterol derivatives [23]. However, BetA-induced apoptosis is not mediated by the Fas receptor, which is activated when membrane fluidity is increased after cellular exposure to methyl-β-cyclodextrin [52]. Suh et al. and Ji et al. have reported that synthesized triterpenoids synergize with TGF-β to induce the expression of TGFβ receptors as well as the activity of the TGF-β-responsive plasminogen activator inhibitor-1 (PAI-1) promoter, prolong Smad phosphorylation and signaling, and activate the promoters of activin and BMP reporter constructs [53, 54]. However, despite all of these observations, an immediate, direct molecular target of triterpenoids in the TGF-β network has yet to be identified [55]. Di Guglielmo et al. [38] and Chen et al. [18, 19, 26] have demonstrated that TGF-β responsiveness is determined by TGF-β receptor partitioning between lipid raft/caveolae-mediated and clathrin-mediated endocytosis. Lipid raft/caveolae-mediated endocytosis facilitates TGF-β degradation, thereby suppressing TGF-β responsiveness. By contrast, clathrin-mediated endocytosis results in Smad2/3-dependent endosomal signaling, thus promoting TGF-β responsiveness. Based on the dominance model for the signal that controls TGF-β partitioning between the 2 distinct endocytosis pathways [18, 19, 26, 36], to the best of our knowledge, this study is the first to propose that BetA increased TGF-β responsiveness through lipid raft disruption and by pushing the TGF-β-TGF-β receptor complex into the non-lipid raft microdomain in the plasma membrane. Several lines of evidence presented herein indicate that BetA is a TGF-β receptor activator that can effectively enhance (Smad2/3-dependent) TGF-β responsiveness in Mv1Lu cells: BetA treatment (1) enhances TGF-β-induced transcriptional activation such as PAI-1, fibronectin, and the collagen promoter, and these “BetA-specific” activations are antagonized by cholesterol treatment; (2) enhances TGF-β-induced signaling such as Smad2 phosphorylation and nuclear translocation; (3) increases TGF-β-induced fibronectin expression in Mv1Lu cells; (4) augments TGF-β-induced growth inhibition in Mv1Lu cells; and (5) most important, the effect of BetA on TGF-β responsiveness is rapid and coordinates with BetA-induced TGF-β receptor translocation from lipid raft/caveolae to non-caveolae on the plasma membrane. The effect starts from 30 min after the incubation of BetA-treated cells, and reaches its maximum effect at 2 h (unpublished results). In this study, we demonstrated that BetA treatment increases the accumulation of TβR-I and TβR-II to non-lipid rafts/non-caveolae (as Complex I) (Fig. 10), resulting in enhanced TGF-β responsiveness. The depletion of cholesterol from the plasma membrane by cholesterol-depleting agents (e.g., nystatin) leads to a decreased formation or destabilization of lipid rafts/caveolae, thereby also increasing the localization of TβR-I and TβR-II (as Complex I) in non-lipid raft microdomains (Fig. 8a). It is possible that a number of these activities are mediated by their ability to affect the structure and function of lipid rafts/caveolae, which are known to modulate signaling mediated by G protein-coupled receptors, receptor tyrosine kinases (EGFR), TGF-β receptors (TβR-I and TβR-II), and possibly others [21, 26, 38, 56–58]. However, in the present study, EGFR membrane microdomain localization remained unchanged after BetA treatment. These results implicate the specificity of BetA treatment in TGF-β responsiveness. In conclusion, this is the first report to show that BetA augments TGF-β signaling by shifting TGF-β receptors from the lipid raft/caveolae to non-caveolae microdomain in the plasma membrane.

## Additional file

Additional file 1: Figure S1. The treatment of BetA does not change the levels of TβR-II, TβR-II, and caveolin-1 in Mv1Lu cells. **Figure S2.** SB431542 inhibits BetA-enhanced TGF-β-induced fibronectin expression in Mv1Lu cells. (PDF 325 kb)

## Abbreviations

TGF-β: transforming growth factor-beta
BetA: betulinic acid (3b, hydroxy-lup-20(29)-en-28-oic acid)
TfR: transferrin receptor
EEA1: early endosome antigen 1
TβR-I: type I TGF-β receptor
TβR-II: type II TGF-β receptor
PAI-1: plasminogen activator inhibitor-1
Mv1Lu cell: mink lung epithelial cell
ALK-5: activin receptor-like kinase
MTT: (3-[4,5-dimethylthiazol-2-yl]-2,5-diphenyl tetrazoliumbromide)
ECL: enhanced chemiluminescence
NFκB: nuclear factor kappa-light-chain-enhancer of activated B cells
PPARγ: peroxisome proliferator-activated receptor gamma
IκBα: nuclear factor of kappa light polypeptide gene enhancer in B-cells inhibitor-alpha
