# Supplementary figures and images for: Betulinic acid enhances TGF-β signaling by altering TGF-β receptors partitioning between lipid-raft/caveolae and non-caveolae membrane microdomains in mink lung epithelial cells
Source: J Biomed Sci. 2016 Feb 27;23:30. doi: 10.1186/s12929-016-0229-4 (PMC4769553; doi:10.1186/s12929-016-0229-4)

Supplemental data, Figure S1

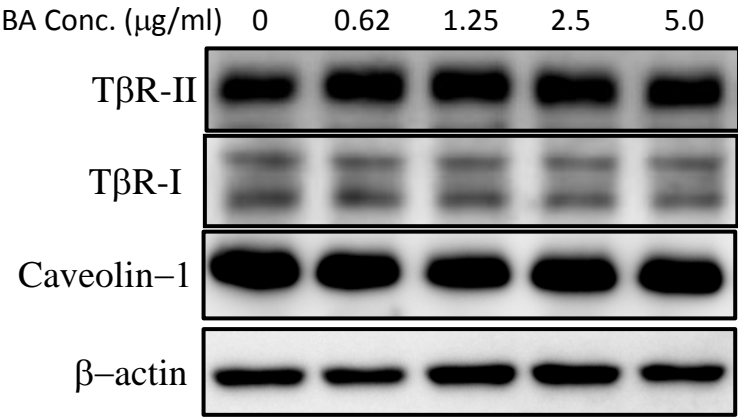

Supplemental data, Figure S2

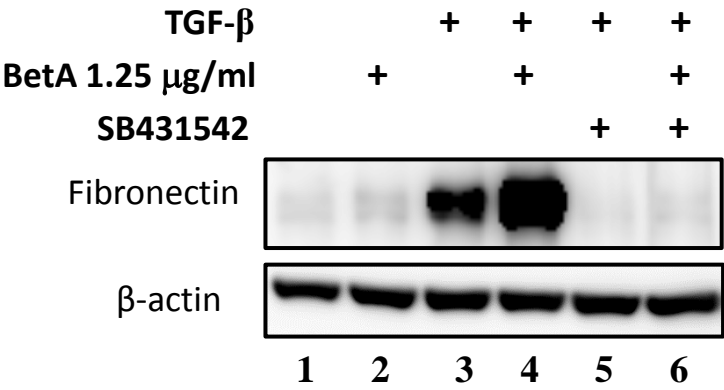

Supplement: Additional file 1: Figure S1. — The treatment of BetA does not change the levels of TβR-II, TβR-II, and caveolin-1 in Mv1Lu cells. Figure S2. SB431542 inhibits BetA-enhanced TGF-β-induced fibronectin expression in Mv1Lu cells. (PDF 325 kb) [file 12929_2016_229_MOESM1_ESM.pdf]
